# “Trust people you’ve never worked with” – A social network visualization of teamwork, cohesion, social support, and mental health in NHS Covid personnel

**DOI:** 10.3389/fpsyg.2024.1293171

**Published:** 2024-02-20

**Authors:** Stefan Schilling, Maria Armaou, Zoe Morrison, Paul Carding, Martin Bricknell, Vincent Connelly

**Affiliations:** ^1^Psychology, Faculty of Health and Life Sciences, University of Exeter, Exeter, United Kingdom; ^2^Department of Psychology, Health and Professional Development, Oxford Brookes University, Oxford, United Kingdom; ^3^School of Health Sciences, University of Nottingham, Nottingham, United Kingdom; ^4^Aberdeen Business School, Robert Gordon University, Aberdeen, United Kingdom; ^5^Oxford Institute of Nursing, Midwifery and Allied Health Research, Oxford Brookes University, Oxford, United Kingdom; ^6^King’s College London, School of Security Studies, London, United Kingdom

**Keywords:** COVID-19, inter-professional, inter-disciplinary, healthcare, teamwork, mental health, leadership, preparedness

## Abstract

**Background:**

The unprecedented rapid re-deployment of healthcare workers from different care pathways into newly created and fluid COVID-19 teams provides a unique opportunity to examine the interaction of many of the established non-technical factors for successful delivery of clinical care and teamwork in healthcare settings. This research paper therefore aims to address these gaps by qualitatively exploring the impact of COVID work throughout the pandemic on permanent and deployed personnel’s experiences, their ability to effectively work together, and the effect of social dynamics (e.g., cohesion, social support) on teamwork and mental health.

**Methods:**

Seventy-five interviews were conducted across the UK between March and December 2021 during wave 2 and 3 of COVID-19 with 75 healthcare workers who were either permanent staff on Intensive Care/High Dependency Units used as COVID wards, had been rapidly deployed to such a ward, or had managed such wards. Work Life Balance was measured using the WLB Scale. Interview transcripts were qualitatively coded and thematic codes were compared using network graph modeling.

**Results:**

Using thematic network analysis, four overarching thematic clusters were found, (1) teamwork, (2) organizational support and management, (3) cohesion and social support, and (4) psychological strain. The study has three main findings. First, the importance of social factors for teamwork and mental health, whereby team identity may influence perceptions of preparedness, collaboration and communication, and impact on the collective appraisal of stressful events and work stressors. Secondly, it demonstrates the positive and negative impact of professional roles and skills on the development of teamwork and team identity. Lastly the study identifies the more pronounced negative impact of COVID work on deployed personnel’s workload, mental health, and career intentions, exacerbated by reduced levels of social support during, and after, their deployment.

**Conclusion:**

The thematic network analysis was able to highlight that many of the traditional factors associated with the successful delivery of patient care were impeded by pandemic constraints, markedly influencing personnel’s ability to work together and cope with pandemic work stressors. In this environment teamwork, delivery of care and staff well-being appear to depend on relational and organizational context, social group membership, and psycho-social skills related to managing team identity. While results hold lessons for personnel selection, training, co-location, and organizational support during and after a pandemic, further research is needed into the differential impact of pandemic deployment on HCWs mental health and teamwork.

## 1 Introduction

The complexity, patient volume, and severity of COVID-19, exacerbated by already existing staff shortages in the healthcare sector, required an unprecedented upscaling of capacity during the peak phases of COVID-19. Hospitals around the world relied on the deployment of nurses, doctors, and allied health professionals to provide relief and support for overwhelmed and understaffed personnel in Intensive Care, Infectious Disease, and High Dependency Units (ICU, IDU, HDU) (Mahendran et al., 2020; Soled et al., 2020; Tannenbaum et al., 2020). As many of those deployed had little to no prior experience or training in intensive, acute, or infectious disease (ID) care, such *ad-hoc* deployment of health-care workers (HCW) into COVID ICUs may have undermined many of the antecedents of inter-professional teamwork in healthcare teams. For example, research has repeatedly found substantial benefits of interprofessional/interdisciplinary (IP/ID) teamwork on staff well-being and social support and was linked to improved integrated care and patient outcomes, patient satisfaction, as well as reduced treatment costs, mortality rates, length of in-patient stay, and clinical error rates (Husebø and Akerjordet, 2016; Ballangrud et al., 2017; Schmutz et al., 2019). However, effective IP/ID teams rely on prior relational coordination and establishment of shared mental models, something that the rapid and often fluid amalgamations of personnel from different professional backgrounds during COVID may not have had.

In addition to the fluid composition of teams, the hazardous environment alongside social distancing, and personal protective equipment (PPE) may have weakened many of the established non-technical factors influencing delivery of standardized care: teamwork, communication, social support, relational coordination, and exacerbated pre-existing occupational identities and spatial–temporal separation with other team-members and leaders (O’Leary et al., 2011; Salas et al., 2016; Dinh et al., 2019; Schilling and Armaou, 2023). A recent review, examining barriers and facilitators for teamwork in IP/ID teams, found that the literature on rapidly deployed personnel in intensive and acute care settings found that little is known about how permanent and rapidly deployed personnel experience teamwork on intensive care wards (Schilling et al., 2022). Likewise, despite socially supportive, and cohesive teams often described as instrumental in countering conflict within work teams, few studies in the review detailed the role of social group membership on IP/ID teamwork.

Additionally, ample research has demonstrated the negative consequences of pandemic work on HCWs mental health and well-being (Vyas et al., 2016; Kisely et al., 2020; Spoorthy et al., 2020), with clinical personnel exhibiting higher rates of mental health problems than the general population, and high prevalence rates of depression (27–40%), anxiety (27–37%), and PTSD (20–49%) in HCWs (Wu et al., 2020; de Sousa et al., 2021; Saragih et al., 2021). For example, a large UK survey of over 6,000 healthcare staff showed not only an increase in probable mental health disorders during the COVID-19 pandemic, but also emphasized the elevated risk of mental health disorders for younger and less experienced nursing staff (Hall et al., 2022). While some studies have examined the lived experience of HCWs during COVID, most of these occurred during or shortly after Wave 1 (Feb. to June 2020 in the UK) (Grailey et al., 2021; Jesuthasan et al., 2021; Manthorpe et al., 2021; Conolly et al., 2022; Kotera et al., 2022; Maben et al., 2022; Stayt et al., 2022), resulting in a scarcity of qualitative evidence from HCWs on the effect beyond Wave 1. Similarly, limited research is available on how social and non-technical factors for care delivery were impacted by COVID-19 guidelines. This research paper therefore aims to address these gaps and expand upon the existing literature by qualitatively exploring 1) how COVID work during the first and second wave was experienced by permanent and deployed personnel, 2) how deployed and permanent staff discussed the impact of such work on their mental health 3) how non-technical factors (e.g., teamwork, communication, cohesion, social support) were influenced by workplace adjustments, and 4) how they consequently developed inter-professional teamwork.

The unprecedented rapid re-deployment of personnel from different care pathways into fluid COVID-19 teams provided a unique opportunity to examine the interaction of many of the established non-technical factors for standardized care while also addressing the impact of COVID work on personal health, family life and career intentions. This study enriches the theoretical understanding of teamwork in healthcare during crises by exploring the difficulties faced by *ad-hoc* and rapidly formed inter-professional personnel in establishing and maintaining many of the non-technical team factors necessary for successful delivery of patient care. By interviewing and comparing both permanent (e.g., ICU/HDU personnel) and deployed personnel from non-intensive care background as well as their leaders the study provides a nuanced overview of the structural, psychological, and organizational issues encountered by such emergent healthcare teams.

## 2 Methods

Our study adopted a qualitative deductive exploratory methodology (Bitektine, 2008; Stebbins, 2011; Casula et al., 2021; Schilling, 2022), aimed at expanding upon the pre-existing theoretical knowledge by exploring the lived experience of HCWs during the COVID-19 pandemic. Considering the methodological difficulties of observing team processes during an active Highly Infectious Disease (HID) outbreak, semi-structured video-interviews were chosen to assess HCWs self-reported experiences, and evaluations of their teamwork with colleagues on COVID-19 wards. Two semi-structured interview guides were developed for: 1) frontline facing staff aimed at exploring HCWs perceptions, motivations, shared beliefs, values, and attitudes towards their group and their leaders during their work in IP/ID COVID-19 frontline teams; and 2) leaders (i.e., Clinical or Nursing Directors, Matrons, Senior Managers) aimed at exploring workforce allocation, ward management practices and unearth potential innovations and best practices (The semi-structured interview guides are Data-sheet 1 and 2). These interview guides were designed based on the results from a systematic review of the available scientific evidence on teamwork in *ad-hoc*, fluid, IP/ID healthcare teams during crisis situations (Schilling et al., 2022) and pilot interviews with medical, nursing, and allied health professionals to gain a preliminary understanding of the issues and experiences faced by HCWs during COVID-19 work.

Interview data were analyzed using a sequential Thematic Network Analysis approach (Pokorny et al., 2018; Schilling, 2022), which used network graph modeling to supplement thematic analysis of qualitative interview data. While most thematic analyses are restricted to summary description of the qualitative data, the utilization of network graph modeling permits the added benefit of exploring the inherent structure between themes in a form that is transparent of the research process and replicable by other researchers, without neglecting the qualitative nature of the data (Bruns, 2012; Steinfeld, 2016; Pokorny et al., 2018; Schilling, 2022). Additionally, by utilizing network metrics, (e.g., weighted degree or modularity), the importance of particular themes, the relationships between themes and the potential thematic clustering of themes can be illustrated and further analyzed by showing consistency of themes across different samples (e.g., deployed vs. permanent personnel). Alongside the “rich description” of the participants voice (Maguire and Delahunt, 2017; Castleberry and Nolen, 2018) which allows some insight into potential pathways, the visualization of the textual data allows for both increased transparency about the analytic process and the differences between participant groups as well as improved reproducibility.

### 2.1 Participants

Eligibility criteria for participation were: 18 years or older and a healthcare worker having worked on or managed a COVID-19 ward. Seventy-five interviews were conducted across the UK by two experienced interviewers (SS, MA) between March 2021 and December 2021 (i.e., at the tail end of the second wave and well into the third wave of COVID-19 in the UK) using online video-chat platforms (Google Meet, Zoom, MS Teams). Interviews lasted, on average, 74 min (ranging from 24 min to 125 min). Participants were recruited through 1) designated NHS research sites participating in the study (*n* = 42), and 2) purposive sampling using UK-wide online social media advertisements and snowball sampling (*n* = 33). Recruitment concluded after the recruitment target deemed necessary for adequate representation of all occupational groups and agreed with participating trusts of 12–20 leaders and 55–70 frontline staff had been met. Thirteen participants were recruited in their capacity as leaders (e.g., Matrons, Clinical or Nursing Directors, Senior Manager) and asked questions from the management interview guide. Of these 6 were working on the frontline in patient-facing roles, and 4 were male. The remaining 62 respondents were frontline patient-facing staff, who were predominantly deployed to Intensive Care and High Dependency Units (*n* = 53%) and other non-specified COVID-19 wards (*n* = 18), these could be wards that had been repurposed to function as COVID-19 isolation wards (e.g., rehabilitation or geriatric wards). These were issued the frontline interview guide. There were 55 participants who reported having been deployed or rotated into a COVID-19 ward, 14 participants remained in their permanent team, and seven of the leaders did neither work nor were deployed to a COVID ward.

Of all participants, 30 were registered nurses, 12 doctors, 20 allied health professionals, and four healthcare assistants, nine were “other” various positions in the wider healthcare team (e.g., administration or managerial roles; See Table 1). A total of 27 participants (36%) were in senior roles (e.g., Medical consultants, senior management or Nursing, Midwifery and Health professions Band 7 and above), with the remainder being grade 2–6 (including junior doctors). Participants were primarily female (*n* = 58, 77%), thus matching the gender imbalance in the NHS workforce (NHS Employers, 2019). Most participants identified as White British or White other (*n* = 63, 84%), with the remaining 12 participants identifying as multiple ethnic (*n* = 6), Black African Caribbean (*n* = 3) or Asian and Asian British (*n* = 3). The low percentage of personnel with minority backgrounds may be a consequence of NHS guidance for black, Asian and minority ethnic (BAME) HCWs to reduce risk of infection following early evidence of disproportionate mortality and morbidity among BAME personnel (NHS England, 2020).

**Table 1:** Overview of participant demographics.

Participant demographics		
Total participants	*N* (Total)	*N* (Deployed)
	75 (100%)	55 (73%)
*Gender*		
Female	58 (77%)	41 (55%)
Male	17 (23%)	1 (<1%)
*Frontline staff vs. Leader*		
Frontline staff	62 (83%)	50 (66%)
Leaders	13 (13%)	5 (6%)
*Seniority*		
Junior (Band 3–6 & jun. Doctor)*	48 (64%)	38 (51%)
Senior (Band 7–8 & Registrar/Consultant)*	27 (36%)	17 (23%)
*Prior intensive/critical care experience*		
NO	27 (36%)	23 (31%)
YES	45 (60%)	32 (43%)
N/A	3 (4%)	
*Occupational group*		
Registered nurse	30 (40%)	22 (29%)
Medical doctor	12 (16%)	9 (12%)
Allied health professional	20 (27%)	15 (20%)
Wider healthcare team/management	8 (11%)	5 (7%)
Nursing or healthcare assistant	4 (5%)	3 (4%)
Physician associate	1 (<1%)	1 (<1%)
Occupational specialty	N (Total)	N (Deployed)
Intensive/Critical/A&E	20 (27%)	11 (16%)
General medicine	20 (27%)	19 (27%)
Infectious, respiratory, hematology	9 (12%)	7 (9%)
Other (e.g., metabolic, pediatric, palliative, skeletal)	12 (16%)	11 (16%)
Other (e.g., sexual, mental, diet, occupational)	10 (13%)	6 (8%)
No Clin Specialty	4 (5%)	1 (<1%)
*Ward location*		
COVID ward	18 (24%)	16 (21%)
Emergency Dept (ED)	7 (9%)	4 (5%)
High dependency unit (HDU)	12 (16%)	10 (13%)
Intensive care unit (ICU)	28 (37%)	21 (28%)
Other	3 (4%)	1 (<1%)
N/A	7 (9%)	
*Total Length of COVID work*		
N/A	14 (19%)	8 (11%)
01–03 months	14 (19%)	13 (17%)
04–07 months	20 (27%)	20 (27%)
08–11 months	4 (5%)	3 (4%)
12–15 months	5 (7%)	2 (3%)
16–19 months	13 (17%)	6 (8%)
19–22 months	5 (7%)	3 (4%)
Ethnicity	N (Total)	N (Deployed)
White (British)	55 (73%)	40 (53%)
White (Other)	8 (11%)	7 (9%)
Mixed/multiple ethnic	6 (8%)	3 (4%)
Black/African/Caribbean	3 (4%)	2 (3%)
Asian/Asian British	3 (4%)	3 (4%)

Of all participants 45 reported prior experience working in intensive, critical, or emergency care environments, with 27 reporting no such experience (three gave no details). Participants’ occupational specialty was predominantly intensive or critical care and general medicine, with nine from non-intensive care specialties with COVID-19 relevant expertise and procedures such as infectious disease, respiratory or hematology. At the time of the interviews, participants had been working in a COVID area for an average of 8.8 months, with the longest duration being 20 months. Most of the short-term exposure on COVID wards were deployed personnel during COVID wave 1 (Feb. to June 2020) or wave 2 (September 2020 to March 2021), while the long-term staff were predominantly qualified permanent ICU staff. Not all staff were deployed during all waves, overall, 47 of the patient-facing staff had experience of working during wave 1, 47 in wave 2, and 11 in wave 3.

### 2.2 Data collection

The semi-structured character of the interviews provided a basis to explore topics identified through systematic review and pilot interviews (e.g., COVID-19, Work and Team Integration, Cohesion, Teamwork, Leadership, Mental Health and Support, Career Implications, Impact on Personal Life and Family). This allowed participants the opportunity to direct the discussion and provide a rich understanding of leadership, teamwork, team bonding, and social support as discussed by both leaders (*N* = 13) and patient-facing frontline staff (*N* = 62) (Creswell et al., 2013). Additionally, some demographic measures were included to allow for subsequent cross-sectional analysis across different occupational groups (e.g., nurses, doctors, and allied health professionals) covering deployment status (e.g., deployed versus permanent staff), ICU experience, specialization, age, and work length. Some standardized survey items were used to assess the level of work life balance (WLB) (Sexton et al., 2017; Schwartz et al., 2019) and common mental disorders (using the GHQ-12) (Goldberg and Hillier, 1979; Goldberg et al., 1997; Anjara et al., 2020) to provide additional context (Creswell et al., 2013) Participation was voluntary following informed consent with interview sessions being audio-recorded, with the audio transcribed verbatim, cleaned and pseudonymized.

### 2.3 Data analysis

The qualitative interviews were analyzed using a Thematic Network Analysis approach (Pokorny et al., 2018; Schilling, 2022), which first investigated the transcripts via thematic analysis, using NVivo (release 1.6.2; Woolf and Silver, 2017; QSR, 2020). Transcripts were double-coded by two experienced coders (SS, MA) using a sequential deductive exploratory coding method, by which we coded the transcripts (a) using the 13 themes identified in our prior systematic literature review (e.g., “shared mental models” (Cannon-Bowers et al., 1993; Salas et al., 2016), “formal communication”, “cohesion” (Schilling et al., 2022) as initial deductive coding guide, which was then extended upon through (b) inductive coding focused on themes emerging from the data, reflective of the topics brought forward by the participants (Braun and Clarke, 2012; Braun and Clarke, 2021), and not previously identified as deductive themes (e.g., “familiarity with tasks”, “inside vs. outside of ward”). To maintain the context and reflect human speech, whereby a speech fragment is discussing several different themes simultaneously, the data was coded en-bloc (e.g., one paragraph) and against all potential codes within that paragraph (e.g., Teamwork, Leadership, Anger and Frustration. Supplementary Table 5). The resulting codes were tested for inter-rater reliability, before the deductive and inductive themes were merged and synthesized to ensure they adequately represented the interviewees’ narrative accounts. The final 80 thematic codes were presented to the study’s advisory board and an expert panel of healthcare professionals who verified and confirmed them.

**Figure 1:**
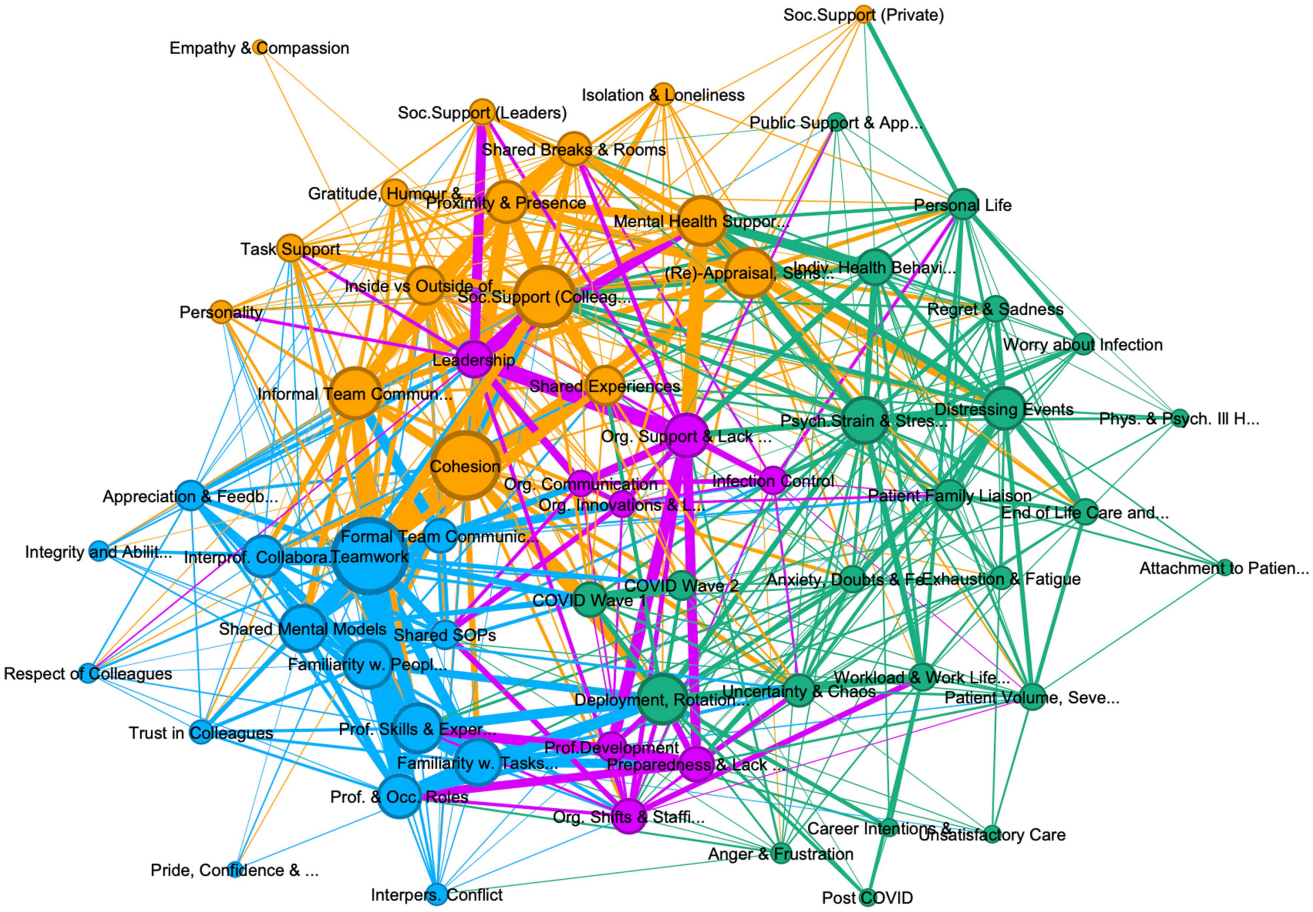
Graph of connections between codes in all personnel, showing 62 codes, with 4 community clusters: (1) teamwork (teal 24%), (2) psychological strain (green, 37%), (3) organization support and management (purple 13%), and (4) cohesion and social support (khaki, 26%). Lift >5, edge weight > 10, graph density: 0.271, size average weighted degree (722.58), and communities by modularity (Modularity: 0.295).

**Figure 2:**
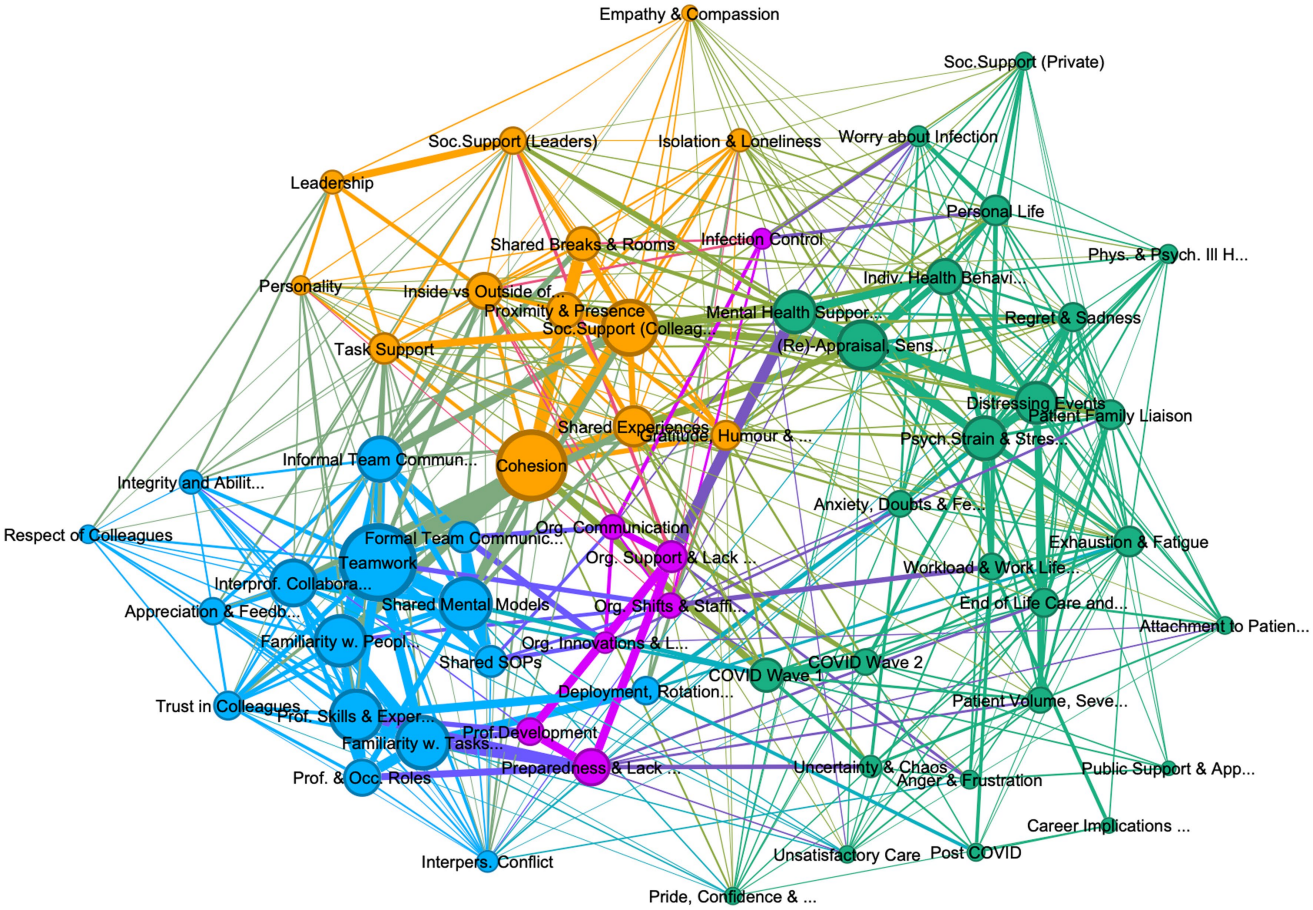
Graph of connections between codes in deployed personnel, showing 62 codes, with 4 community clusters: (1) teamwork (teal 26%), (2) psychological strain (green, 42%), (3) organization support and management (purple 11%), and (4) cohesion and social support (khaki, 21%). Lift >5, edge weight > 10, graph density: 0.241, size average weighted degree (414.6), and communities by modularity (Modularity: 0.384).

**Figure 3:**
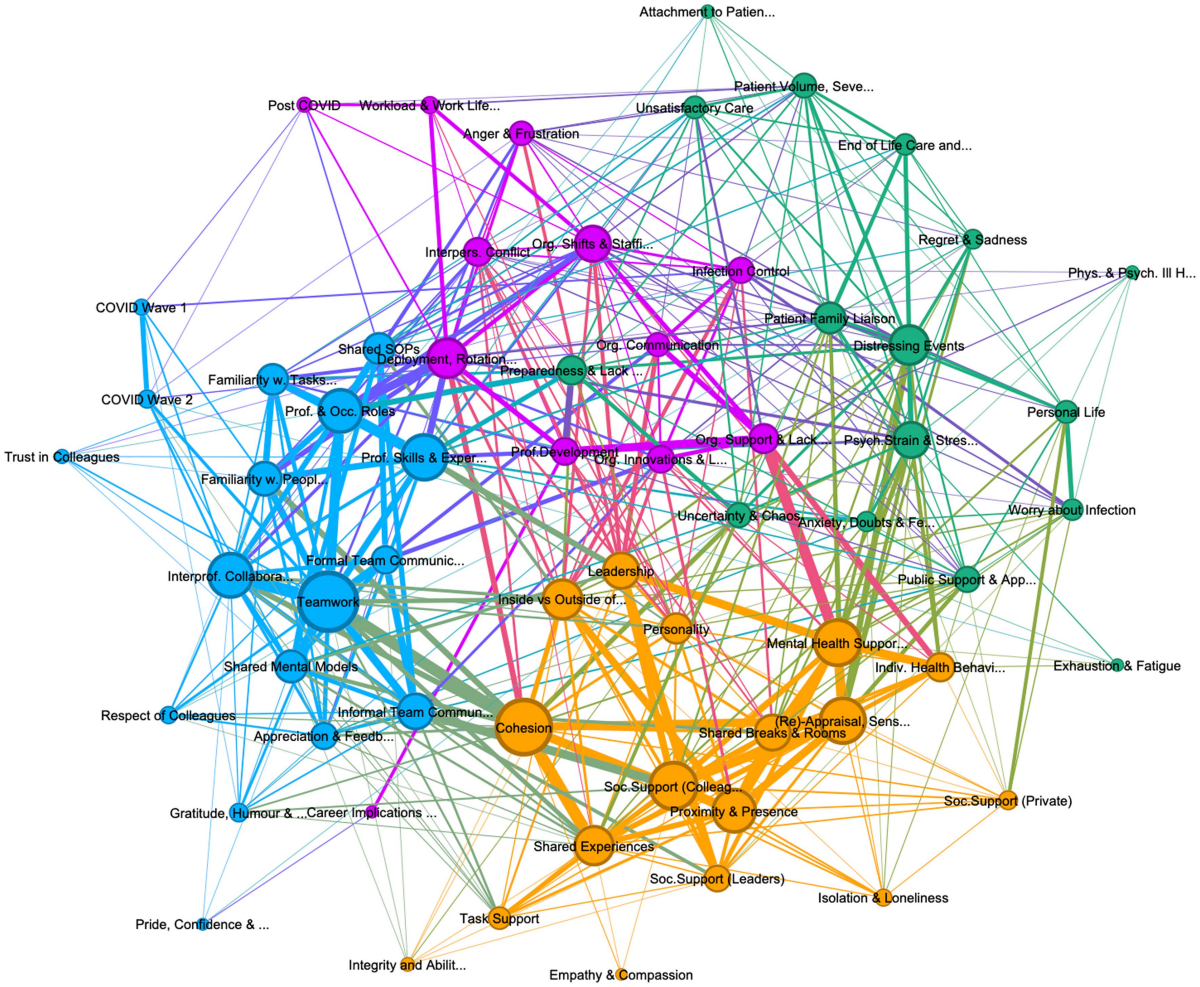
Graph of connections between codes permanent personnel, showing 62 codes, with 4 community clusters: (1) teamwork (teal 27%), (2) psychological strain (green, 27%), (3) organization support and management (purple 19%), and (4) cohesion and social support (khaki, 27%). Lift >8, edge weight > 4, graph density: 0.235, size average weighted degree (141.09), and communities by modularity (Modularity: 0.331).

In a second step – once thematic coding had been completed – the coded data was further explored using network modeling. A matrix table (see Masterfile), consisting of all thematic codes and the number of shared references between them (e.g., Teamwork and Cohesion share 200 references) was extracted from NVivo. The resulting table was formatted with the number of references shared between codes formatted as edge-weight and uploaded into a network analysis and exploration software program [Gephi release 0.10, (Cherven, 2015)]. Edges in the network were undirected and created based on code co-occurrence in the same paragraph. The resulting weighted network was filtered by applying edge-weight and the association rule measure “lift” to minimize noise, with the lift and edge-weight threshold determined using the elbow method for cluster detection (Braesemann, 2019; Humaira and Rasyidah, 2020; Shi et al., 2021). The resulting graphs (Figures 1–3) were visualized in Gephi with the Force Atlas 2 graph layout algorithm (Fruchterman and Reingold, 1991; Gemici and Vashevko, 2018) and using the Leiden modularity algorithm to determine communities within the data (Blondel et al., 2008; Drieger, 2013; Ji et al., 2015; Kang et al., 2017). The visualization of the graph is determined by 1) the importance of particular constructs, represented by the centrality and distance of the node from the center; 2) the size of the nodes based on the number of shared references with other codes (weighted degree); 3) the number of shared references between two codes represented by the thickness and color of the connections between codes (i.e., edges); and 4) the community structure of the codes, representing which cluster of codes are more closely related to each other (modularity clusters). A modularity comparison identified whether thematic codes consistently appeared in specific clusters across different participant groups see Supplementary Table 4.

To provide context about participants’ work, group differences of perceived Work-Life Balance Impairment (WLB) and Common Mental Disorders (GHQ-12) between deployed and permanent personnel, those with and without ICU experience, and between junior and senior staff were analyzed using an independent *t*-test. Due to the small sample size no further statistical analysis – which could allow for generalizable correlational results – were conducted.

### 2.4 Ethical considerations

Ethical approval was gained from the Oxford Brookes University Research Ethics Committee (UREC# E20025) and regulatory approval was gained through the UK Health Research Authority (IRAS# 294169). All participants provided written informed consent prior to being contacted for the interview, and verbal consent at the beginning of the interview.

## 3 Results

Impaired work life balance was experienced by 51% of participants on two or more days per week (*M* = 2.02, SD = 0.505) with nutrition, coming home late from work, and difficulty sleeping showing the highest reported impairment. The 14 permanent participants (*M* = 1.74, SD = 0.375) compared to the 55 deployed participants (*M* = 2.07, SD = 0.507) showed significantly lower WLB scores, *t*(66) = 2.21, *p* = 0.026, *d* = 0.68, indicating less impaired work-life balance per week. Similarly, participants with ICU experience (*N* = 45, *M* = 1.92, SD = 0.491), showed statistically significant lower rates of WLB scores than personnel without any ICU experience (*N* = 27, *M* = 2.18, SD 0.505), *t*(70) = 2.14, *p* = 0.036, *d* = 0.531. Interestingly, those who identified as junior (i.e., junior doctors and band 6 and below) showed significantly lower levels of WLB scores (*N* = 48, *M* = 1.93, *SD* = 0.417), than participants with band 7 or higher and consultants (*N* = 25, *M* = 2.19, SD = 0.616), *t*(71) =2.12, *p* = 0.038, *d* = 0.522 (see Table 2). Higher WLB scores were stable in deployed and those without ICU experience across different occupational specialties (i.e., Intensive Care, Infectious Disease and Respiratory, General Medicine; Pediatric and Palliative, and Mental Health, Dietician, Occupational, Sexual Health). Sixteen participants scored above the threshold of 3 on the GHQ-12 (GHQ scoring), indicating potential common mental disorders in 21.3% of participants. No statistically significant differences were found between leaders and frontline staff and between those deployed and permanent.

**Table 2:** Group results for work life balance scores (WLB) for permanent vs. deployed participants and participants with ICU and no ICU experience.

	Group	*N*	Mean	SD	SE
WLB Scale	Permanent	14	1.74	0.375	0.1
	Deployed	55	2.07	0.507	0.0684
	ICU Exp	45	1.92	0.49	0.073
	Non-ICU Exp	27	2.19	0.505	0.097
	Junior	48	1.93	0.417	0.06
	Senior	25	2.19	0.616	0.123

### 3.1 Thematic findings

The thematic analysis of the interviews identified 80 codes, of which 18 were excluded as subsidiary codes. The interactions between the remaining 62 codes were visually explored in three graphs, one containing all references from all personnel interviewed, including leaders (Figure 1), the second containing only deployed personnel (see Figure 2) and one containing only permanent personnel (see Figure 3). Modularity calculation using the Leiden algorithm found evidence of four thematic community clusters within the graphs, namely 1) Teamwork (teal), 2) Organizational Support & Management (purple), 3) Cohesion & Social Support (khaki), and 4) Psychological Strain (green) (Examples for the themes can be Supplementary Table 5). The clusters showed a high degree of consistency across the three graphs, with 65% of codes in the cohesion cluster occurring in this cluster in all three graphs, followed by teamwork (52%), psychological strain (48%), and organizational support and management (30%) (see Table 3) An overview of the codes with corresponding number of references, weighted degree, their clusters in each of the three graphs, can be Supplementary Table 4.

**Table 3:** Overlap of codes within the four identified clusters.

	Teamwork in COVID wards	Cohesion and social support	Organization support and management	Psychological strain
# of Codes occurring in all 3 graphs	12	11	6	15
% of overlap	52%	65%	30%	48%
# of Codes occurring in 2 graphs	4	5	1	7
% of overlap	17%	29%	5%	27%
# of Codes occurring in 1 graph	4	3	7	6
% of overlap	17%	18%	35%	23%
Total Codes in Cluster	20	19	14	28

#### 3.1.1 Thematic cluster 1: teamwork in COVID wards

Represented by the thickness of the connecting edges, the graph emphasizes that individual references discussing teamwork most consistently included procedural (e.g., shared mental models and SOPs), professional (skills and experience, professional roles), relational (familiarity with colleagues and their skills), and communication codes (formal and informal team communication). Emotional codes such as appreciation and feedback, trust in colleagues, and respect for colleagues were also associated with this cluster. The adjacency of shared mental models, team communication, and familiarity with colleagues in the graph highlights that effective communication, a common understanding of goals and responsibilities, and common procedures are most closely aligned to the description and perception of teamwork and interprofessional collaboration across the sample. For example, differing communication standards between intensive care and deployed personnel, increased noise levels on the wards, and usage of PPE undermined communication, and reportedly led to miscommunication within the team.

Considering these difficulties, the interviews frequently emphasized the importance of communication as critical for the development of teamwork and shared mental models. Specifically in the first wave, where clinical guidance and SOPs for COVID were rare, formal communication during handovers and ward rounds, using bedside clinical documentation, or virtual communication between IP/ID team-members, were crucial for the development of shared mental models and the allocation of responsibilities and tasks, involving a range of IP/ID professional skills and experiences. While the graph of the wider sample (Figure 1), shows informal communication to be in the cohesion cluster, both the deployed and permanent graphs, show informal team communication as contributing to teamwork, suggesting that peers consider informal communication (e.g., check up on each other, communicate breaks, or provide brief moments of respite) as important as formal communication procedures. In fact, many participants emphasized the importance of such informal communication due to reductions in social interactions outside of the ward, or due to social distancing guidelines on the ward. Relatedly, familiarity with colleagues was highly important to staff members’ perception of teamwork, with permanent personnel in large hospital trusts more likely to accentuate a lack of familiarity with colleagues as impeding teamwork, due to higher fluctuation of staff, while participants from smaller NHS trusts, reported lower disruption to their teams, but – in some cases – higher levels of difficulty integrating deployed staff into long-standing fixed teams.

Importantly, the graph also highlights that besides the codes in the teamwork cluster, other codes outside of the immediate cluster impact upon teamwork. For example, teamwork shares a lot of references with cohesion, indicated by the closeness of these codes to each other and the thickness of the edge between them. The adjacency of these codes to each other and the overlap of some of the surrounding codes (e.g., informal communication, familiarity, shared experiences) underscores the importance of social factors and camaraderie on effective teamwork. Descriptions such as “*collective identity [as] one team [with] the same goal of treat[ing] patients and looking after each other”* (RES027) were very common, highlighting the convergence of teamwork and social care of colleagues.

Across the entire sample, professional and occupational roles, prior professional skills, and familiarity with COVID specific tasks (e.g., ventilation, turning patients with IV lines, giving medication) were emphasized as contributing factors for effective teamwork. In particular, trust in colleagues was based on both having a shared understanding of the team’s goals and everyone’s role within it, and on the perceived skills and abilities deduced from someone’s occupational category (e.g., “oncology personnel can handle IV lines and cytoxic medications”, “respiratory physios can support CPAP training”). However, many participants reported a higher degree of teamwork within the IP/ID team in the first wave, precisely because of a “blurring” of these roles and a reduction of medical hierarchies, whereby doctors, AHPs and nurses often shared tasks that normally are provided by only one occupational group (e.g., patient care, proning/turning of patients).

This reduction of hierarchies and blurring of roles was often attributed to the uncertainty of wave 1, in which the lack of clinical guidance flattened hierarchies and elevated personnel with COVID-19 relevant skill sets. For example, nurses or AHPs were included into bedside rounds and those with respiratory expertise were given additional responsibilities training up personnel on CPAP ventilation and co-developing local clinical procedures. Nevertheless, many of the references discussing professional roles or skills were also related to anger and frustrations, interpersonal conflict, and career intentions. For example, ICU/HDU personnel routinely reported that lack of experience by deployed staff undermined teamwork, trust, and communication, and increased the responsibility and workload of experienced staff. In contrast, participants across the sample underscored that showing willingness to learn, asking questions, and receiving feedback from experienced colleagues as well as being able to admit lack of knowledge were effective ways to increase teamwork by establishing common procedures and enhancing integration into the team.

With shared uncertainty providing a unifying experience, interpersonal conflict reportedly occurred less in the first wave, but increased in the second wave with the dissemination of established clinical management guidelines, the need for more personnel, and refined Infection Prevention and Control (IPC) measures. Such measures reportedly deterred large handovers or bed-side rounds involving the entire IP/ID team and led to more social distancing in staff and break rooms, and a return of medical hierarchies. For example, many nurses and AHPs who had perceived working relatively equally with doctors in the first wave, spoke of a perceived loss of responsibility and involvement in the second wave, and personnel reported not being able to attend meetings or break rooms that had now been segregated by occupation. Two frequently cited issues for conflict were the inability to fulfill role expectations (for example deployed staff being admonished for not knowing how to do medication rounds or read blood gasses), or overconfidence in a task performed without formal training, increasing the risk for mistakes. Much of such conflict appears most strongly linked to professional roles and skills, and familiarity with tasks, with participants reporting that misperceptions about someone’s responsibilities or false expectations about their occupational skills were often at the root of interpersonal conflict. Particularly in the second wave’s surge in personnel, some team-leaders highlighted the importance of short introductions at the beginning of every shift to identify everyone’s professional skills and abilities and thus appropriately assign responsibilities and patients based on someone’s experience.

**Figure 4:**
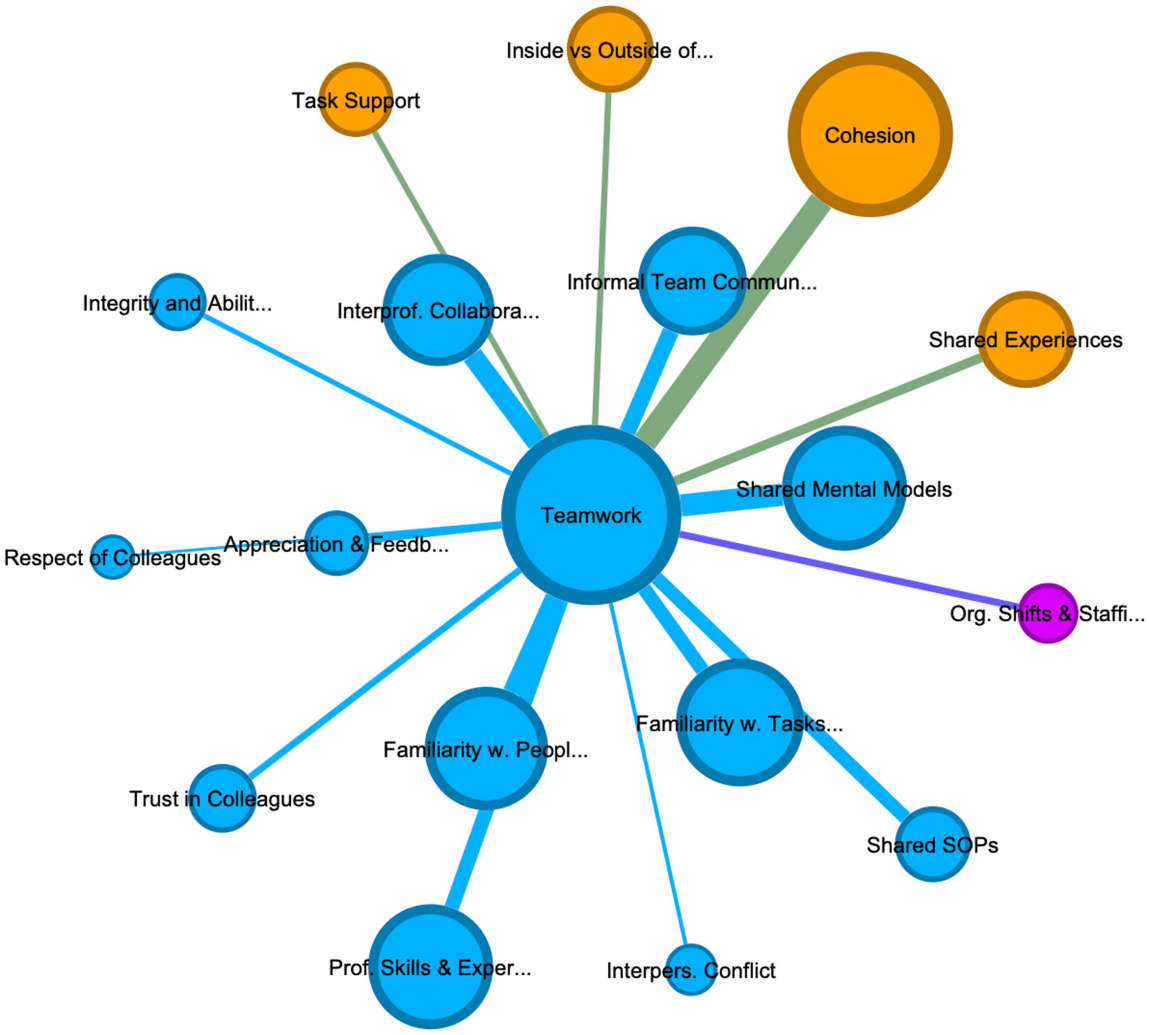
Teamwork in deployed personnel: this graph displays the codes most connected to Teamwork in deployed personnel, by filtering Figure 2 using an ego network to only display codes connected to Teamwork. Additionally, a Fruchterman Reingold layout algorithm was applied to further highlight, through adjacency to the code teamwork, which codes share the most references with teamwork in this group.

**Figure 5:**
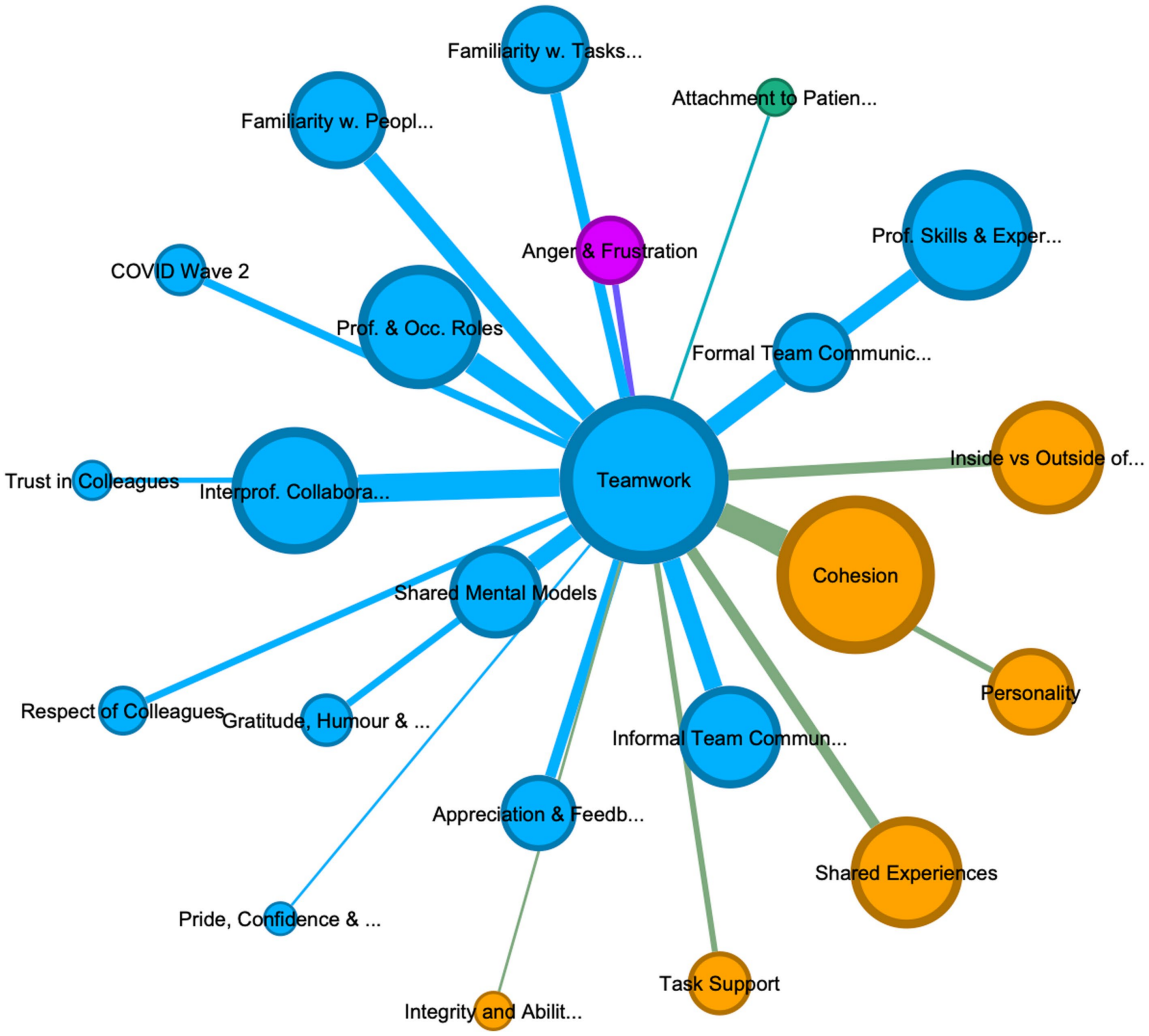
Teamwork in permanent personnel: this graph displays the codes most connected to Teamwork in deployed personnel, by filtering Figure 3 using an ego network to only display codes connected to Teamwork. Additionally, a Fruchterman Reingold layout algorithm was applied to further highlight, through adjacency to the code teamwork, which codes share the most references with teamwork in this group.

It is worth noting the differences between deployed and permanent staff’s description of teamwork. This difference is best highlighted by comparing Figures 4, 5, which depict the codes most closely linked to teamwork in deployed and permanent personnel, respectively. It highlights that while for both groups’ professional skills and experiences, interprofessional collaboration, informal team communication, and cohesion are linked to teamwork in familiarity with both people and tasks as well as appreciation and feedback play a stronger role in deployed personnel, with interpersonal conflict occurring in the teamwork cluster in deployed personnel (Figure 2). This could suggest that rapid integration of deployed individuals into a team, alongside creating familiarity with other personnel and important tasks is particularly important to achieve team working and to reduce conflict.

#### 3.1.2 Thematic cluster 2: cohesion and social support

The cohesion and social support cluster was characterized by a close interaction between codes denoting physical closeness (i.e., proximity, being inside verses outside of the ward, shared breaks and staff rooms), assistance (e.g., task and social support provided by colleagues, leaders or family, and friends), evaluation (e.g., shared experiences, re-appraisal, and sense-making) and emotions (such as gratitude and humor, empathy and compassion, isolation, and loneliness). The graph highlights how often references discussing cohesion or social support occurred alongside comments about shared experiences or being inside the ward as opposed to outside. Cohesion, for many participants appears to be bound intimately to the shared experience of “being in it together,” which determined not only ingroup status, but also provided a basis for emotive evaluation and social support. For example, for many participants in wave 1 the uncertainty of COVID-19, alongside the blurring of roles created a collective identity of being on the frontline of COVID-19 and provided a basis for social support, task support, (i.e., helping colleagues with practical tasks) as well as increased care for the welfare of colleagues. Many participants were thankful for being actively involved in the pandemic response as it allowed for social interactions with colleagues, while everyone else was required to isolate. This was particularly strong in the first wave and for junior and single staff without established social ties outside of work, for whom colleagues on the ward provided an important – and sometimes the only – protective factor against the isolation and loneliness many of them felt outside of work hours during the lockdowns. While descriptions of shared experiences in wave 1 were sometimes even joyful, these changed in the second wave to focus more on distressing events and hardship.

In the graphs (Figures 1–3) for all personnel and permanent personnel, team cohesion and social support is closely tied to mental health support and re-appraisal suggesting a protective function against many of the stressful events and experiences during their COVID work (also compare Figure 1 in Supplementary information). Colleagues were reported to be crucial in providing social support by making sense of individual events on the wards and the pandemic in general, venting about distressing moments, and seeking reassurance about adequate patient care. Seeking reassurance appeared to be most pronounced in deployed personnel with no intensive care experience or junior personnel, who reported struggling with self-doubt or anxiety more often. One participant deployed in wave 2 described it as *“starting to read a book from the middle”* (RES009), where her permanent colleagues explained what had happened in wave 1 and provided reassurance and meaning about the high mortality rate and patient distress. Considering this link between social support, appraisal and mental health, most interviewees pronounced the importance of keeping mental health support within the team (e.g., through mutual peer support sessions), with many making comments like *“the support from other nurses was kind of sufficient for me”* (RES008). Such peer support was reportedly less stigmatized and allowed individuals to make sense of their experiences with those that “had been there” with them.

However, the analysis would indicate that the protective link between team cohesion, social support and mental health relies on stable team-membership, which deployed personnel often were not privy to and correspondingly they reported more difficulties in accessing social support. This is seen in deployed personnel in Figure 2, where re-appraisal and mental health support occur in the patient care and stress cluster. For example, during deployment some reported feeling unable to access support in neither the COVID ward nor their routine place of work, due to their transient status. Many described not being able to discuss their COVID experiences with colleagues upon their return to their old position. Those who were deployed individually felt isolated from colleagues while HCWs who had deployed as a group reported feeling separate from colleagues who had not deployed. It appears that poor cohesion and social support impacts on deployed staff well-being, but also that deployed personnel may be more reliant on organizational efforts to counteract loneliness and negative mental health consequences.

Informal communication – most commonly occurring during breaks or handovers – appeared to be highly important for team cohesion and feeling supported by colleagues. Many participants described the social support arising from moments where colleagues or leaders enquired about one’s welfare, offered a cup of tea, enjoyed happy moments with sometimes dark humor, or the opportunity to sit down and reflect or vent. Such interactions with colleagues were labeled as not only facilitating emotional regulation, but several participants also discussed the importance of colleagues in making sense of their experience. Interestingly, collegial welfare enquiries and corresponding attempts to support each other were reported as more sincere during wave 1, showing a decline during wave 2 with increased fatigue and exhaustion setting in. Similarly, with increased social distancing during the second wave, social isolation and loneliness encroached on cohesion as personnel spent breaks socially distanced or alone and meetings outside of work were further curtailed.

#### 3.1.3 Thematic cluster 3: organizational support and management

Interviewed HCWs portrayed a range of organizational factors pertaining to the day-to-day management of COVID wards and the provision of organizational support, influenced by shift and staffing decisions, organizational communication, IPC measures and professional development. Organizational support was seen by participants as both a resource and a challenge, which was associated with psychological stress and mental health and impacted teamwork. For example, lack of opportunities for professional development, disorganized staff allocation and management, and lack of common break rooms not only impeded information exchange but were consistently mentioned as impacting teamwork. Likewise, some occupational health and well-being support mechanisms introduced by NHS trusts during the pandemic were perceived as well-meaning but problematic by the frontline staff interviewed. For example, many described being unable to attend yoga or mindfulness classes, which tended to occur during their shifts. On the other hand, practical organizational innovations to improve IP/ID relations and communication, such as skills stickers or badges on HCWs PPE, use of iPads or videocall software, improvements to electronic health records, or designated communal rest areas and fixed intra-group debriefs were highly valued.

Organizational communication was reportedly an issue. Specifically, deployed personnel felt frustrated by staffing decisions, commenting on having been deployed or redeployed with little warning, or returning to their routine wards without decompression periods. Constant changes to clinical guidelines, shifting IPC instructions or unclear communication channels were also highlighted as impeding the day-today processes on the wards.

Considering the often very rapid deployment of HCWs, many not having intensive care experience, references about deployment were intimately tied to both technical and psychological preparedness. For example, participants with prior intensive care experience reported a high level of technical preparedness, while those without highlighted a lack of technical training prior to their wave 1 deployment. This changed somewhat in wave 2 with an increase of clinical guidelines and online courses. Nevertheless, almost all participants felt unprepared for the psychological aspects of working with COVID patients, (i.e., the uncertainty and chaos of COVID wards, the patient volume, symptom severity, patient deterioration, distress, and mortality). This suggests that lack of preparedness was an important contributor to psychological stress and in many cases was associated with lack of organizational support. Considering that permanent members discussed preparedness in terms of psychological rather than technical readiness, this code occurs in the stressor cluster in Figure 3. While personnel with prior ICU experience portrayed working on COVID wards as their job or a responsibility to their fellow HCWs, several deployed participants commented on the involuntary nature of their deployment.

While leadership occurs in the organizational cluster in the all personnel graph (which includes leaders, see Figure 1), in both the deployed and permanent participants it occurs in the cohesion cluster, highlighting the ambivalent character of leadership for frontline staff vs. organizational leaders. For example, staff on the wards routinely commented on leadership being performed by near peers, such as junior nurses with critical, intensive, respiratory, or infectious disease expertise (Band 5 and 6), who were allocated leadership over deployed staff termed “bedside buddies” and were thus able to support them on difficult or unfamiliar tasks. Leader legitimacy was closely associated with physical presence, with prior leaders (e.g., matrons, consultants, or ward managers) reportedly losing legitimacy if they were not present on the ward and *“seeing for themselves.”* Specifically in newly opened COVID wards leadership was depicted as shared between different staff members taking on different roles and responsibilities in leading the teams. However, leaders themselves often portrayed their role as having to mitigate a lack of organizational support and supporting staff members mental health and welfare. This stretching of leadership to span social, team-managerial, and organizational functions is also visible in the leadership behaviors described across the sample, e.g., be visible and approachable, lead by example and set the team climate, provide support, check up on staff and “have their back,” provide role clarity and guidance, and assign responsibilities. The range of these leadership behaviors – as well as the adjacency of leadership to other codes such as cohesion, social support and being inside vs. outside the ward (see Figure 1) – highlight why leadership does not fall neatly in one cluster but suggests that during crisis leadership across hierarchical levels is more nuanced.

#### 3.1.4 Thematic cluster 4: psychological strain and stressors

The final cluster is characterized by HCWs reports of psychological strain caused by distressing events, patient care duties, workload, deployment, personal life, and public support which resulted in a range of emotional, physical, and professional responses. Across the sample, the codes which share the most references with psychological strain were patient care tasks and experiences, often labelled as traumatic or distressing. Specifically, patient death related incidents such as informing patients’ families or facilitating last conversations between patients and their families over phones or iPad, alongside witnessing the quick deterioration of patients, were reportedly most problematic (see Figure 1 in Supplementary information) Participants often commented on their disbelief in the first wave at patients’ unprecedented symptom severity and their quick deterioration. While permanent staff and those deployed in wave 1 became used to these symptoms, they reported their shock at the sheer patient volume and the young age of those dying in the second wave. For many staff members, therefore, end of life care and witnessing patients’ distress was problematic, both due to losing unprecedented number of patients – many of their own age – but also because of the level of their distress and the inability to provide patient care in line with their training or professional standards. Many, therefore, discussed perceptions of providing unsatisfactory care due to time, staffing or resource constraints (e.g., not knowing how to treat patients, not having enough oxygen for ventilation, not being able to provide personal care due to patient volume). Finally, some participants, especially those from A&E or ICUs reported increased attachment to patients due to longer hospitalization, making coping with patients’ death harder, and recovery also becoming more meaningful. Interestingly, a lot of participants– even experienced intensive/critical care, and infectious disease staff – would point out that patient care tasks and experiences were something that they were not prepared for. As such, many of these references co-occur with discussions about anxiety and personal doubts, sadness and regret, anger and frustrations, and the negative impact on their own physical and psychological health. Nevertheless, some participants described becoming numb to these experiences, highlighting a gradual normalization to mortality rates, patient distress, and traumatic experiences.

Another repeatedly cited issue related to psychological strain was the sheer workload experienced during COVID work. Although many participants discussed the impact of workload, there appeared to be a more pronounced negative effect of workload on mental health in deployed than permanent personnel. While the latter benefitted from a drop in patients between waves, allowing for some short-term respite, deployed personnel, especially those that returned to their routine positions, reported increased workload due to the backlog in elective treatments. For example, a range of deployed participants on return to routine working described a substantial worsening of symptom patterns in their routine patients (e.g., diabetes, arthritis, cancer), due to not being treated during the lockdowns. More senior deployed personnel, or those in an administrative role, often struggled with the dual pressures of working on COVID wards and supporting their normal team.

The increased stress and workload in deployed personnel appears related to career implications, as many deployed participants depicted these in terms of a re-evaluation of their role after COVID work (e.g., moving into a non-clinical role), alongside career setbacks, such as losing out on important routine rotations or development opportunities. It is important to note that some deployed personnel also perceived their work on these wards as a source of pride, leading to more confidence in their abilities, with a few participants even deciding to retrain as intensivists. One participant, inspired by the camaraderie of wave 1 to retrain as ICU nurse, voiced regret over her decision to when faced with the second wave’s increased stress and exhaustion. The differential impact on workload and career implications is clearly visible by the closeness of these two codes to psychological strain in deployed personnel (Figure 2), compared to permanent personnel, where both codes are associated with codes in the organizational cluster (Figure 3). Correspondingly, permanent staff portrayed career implications in terms of both renewed commitment to their role as intensivist or career progression (i.e., advancing skill sets, changing bands). Interestingly, many junior doctors reported losing out on routine rotations or career opportunities.

Besides these job-internal stressors, most participants reported their COVID work had an impact on their private life. For example, many participants – specifically single, female HCWs – discussed the negative impact on care responsibilities for children or parents as well as the inability to visit friends or family, utilize leisure activities, or access social support outside of work. This was particularly pronounced where family members or friends did not work in healthcare or blue light services and were perceived to “*not know what it’s like,”* resulting in adding additional burden. Interestingly, frustrations and dissatisfaction with public support became a more frequent theme during data collection, with many participants commenting on a reduction of public support in the second wave compared to the first and voicing anger about nonadherence to COVID guidelines and dissatisfaction with the hero dialog exemplified by the public clapping on Thursdays. Many of these comments were made alongside remarks about increased levels of exhaustion and fatigue, suggesting that job-external factors, such as private demands or fading public and governmental support, may impact upon levels of burnout and fatigue in healthcare personnel.

To actively counteract the negative effects on their mental health, participants routinely discussed individual health behaviors for coping with difficult situations, which they developed during the lockdowns. These included for example increased mental health awareness and self-care, seeking help from colleagues, family, and leaders, or seeking professional help from occupational health and psychological services. While the latter were utilized by some, who reported being diagnosed for burnout, PTSD or depression, the emphasis for most participants was on team-internal solutions. In a few instances, where psychological personnel were embedded into wards (e.g., taking on family liaison roles) participants were more likely to report “opening up” about difficult moments than in 1-on-1 counseling, which was often portrayed as less helpful than group sessions with colleagues, due to counselors perceived as not knowing what it was like to work on COVID wards.

## 4 Discussion

This study investigated how deployed or permanent IP/ID personnel working on COVID wards experienced their COVID work and the described impact on their mental health, how permanent and deployed personnel discussed their teamwork, and whether non-technical factors for healthcare delivery (e.g., teamwork, communication, cohesion, social support) were influenced by workplace adjustments and social dynamics within the team. The semi-structured interviews with 75 HCWs, from different occupational background who had been either working (as deployed or permanent staff) on NHS wards treating COVID patients or had managed such wards, were analyzed using thematic coding of transcripts supplemented by a network analysis of the resulting relationships. The thematic network analysis was able to identify four thematic clusters in the data set pertaining to permanent and deployed personnel’s experience of their COVID work, namely 1) Teamwork; 2) Organizational Support and Management; 3) Cohesion and Social Support; and 4) Psychological Strain. Importantly, the adjacency of some codes from neighboring clusters (e.g., cohesion and teamwork) in the graph suggests that the clusters cannot be seen in isolation, but rather that participants frequently discuss these codes within the same reference. While these four thematic clusters are reminiscent of the thematic communities unearthed in our prior systematic review (Schilling et al., 2022 Plos One) suggesting that the literature on teamwork can account for many of the issues discussed by healthcare staff during COVID-19 - it is noteworthy that some of the themes and interactions arising from our interviews received limited exploration in the literature to date. The discussion will consider some of these interactions across community clusters.

### 4.1 Importance of social relations for teamwork and mental health

The analysis demonstrates that social dynamics within the team (i.e., cohesion, social support, proximity, collective appraisal) were pivotal for participants’ description of both teamwork and mental health. Across the sample, descriptions of effective teamwork frequently discussed operational and professional aspects of their work alongside their shared experience and physical proximity of being inside the ward, and the social support they received from colleagues. Cohesion and social support, based on the recognition of “*being in it together”*, also appear to be important protective factors for many, and for most junior personnel seemingly the only one, by aiding the alleviation of stress and making sense of difficult events (Schug et al., 2021). Nevertheless, our study also highlighted that bonds between HCWs as being based on the shared experiences of being on the frontline – a much-reported finding from studies in wave 1 (Jesuthasan et al., 2021; Manthorpe et al., 2021; Conolly et al., 2022; Kotera et al., 2022; Maben et al., 2022) – appeared much more difficult to maintain during the second wave. Echoing the results from a recent study in two U.S. primary care clinics (Lim et al., 2021) it appears that the organizational, and spatial changes, due to increased infection control measures (e.g., social distancing, single occupancy break rooms, or virtual meetings in lieu of large handovers) undermined access to many of these important social resources and thus exacerbated loneliness and isolation. The discussion around public support, alongside the negative impact on personnel’s private life (e.g., changes in care responsibilities, decreased leisure activities, lack of social support), further suggests that for many participants job external factors, may have further contributed to increased levels of burnout and fatigue, and warrants more research.

Our study supports research on the importance of group membership for mental health (Cruwys et al., 2013; Haslam et al., 2019; Bentley et al., 2022) by submitting that many participants described the collective identity as frontline personnel as a protective factor from COVID stressors and distressing events. However, further research is needed to assess the impact of social attraction to the COVID team on the ability to cope with stressors and distressing events experienced during their COVID work. Furthermore, the results hint that older, and more experienced participants with established social circles outside of work were likely more protected from job demands and stressors than junior personnel precisely because they had more group memberships (Cruwys et al., 2013; Steffens et al., 2016; Jetten et al., 2022; Van Dick et al., 2023). Alongside the finding that increased social distancing guidelines in wave 2 increased feelings of social isolation this could suggest that the higher risk for mental health problems in younger and more junior personnel during COVID-19 (Khajuria et al., 2021; Frenkel et al., 2022; Hall et al., 2022) may result from a lack of other avenues of social support (Sani et al., 2015; Steffens et al., 2016). While these relationships need to be further assessed, the results may indicate a potential pathway to decrease elevated risks of mental health problems in more junior personnel (Hall et al., 2022) through measures which increase cohesion and social support. For example, mutual team-based support groups (e.g., Schwartz rounds) may provide safe spaces to share emotional and moral impact of work events while creating shared experiences and shared commonality with colleagues (Dawson et al., 2021; Maben et al., 2021).

### 4.2 Managing interprofessional dynamics and identity to increase teamwork and reduce conflict

The study highlights that effective teamwork in COVID wards was consistently linked to IP/ID dynamics such as team-members differing professional roles and skills, and technical familiarity. The perceived increase of teamwork in the first wave was often attributed to an absence of such dynamics as the general uncertainty and lack of clinical guidance flattened medical hierarchies and led to a blurring of occupational roles and the elevation of personnel with COVID-19 relevant skill sets irrespective of professional background. The subsequent introduction of enhanced clinical guidelines and a surge of personnel during the second wave reportedly restored prior medical hierarchies which again led to a perceived decrease in teamwork. This “slipping into hierarchies” (Dit Dariel, 2018) and the corresponding categorical misperceptions about responsibilities and occupational expectations also appears to be at the root of much of the reported interpersonal conflict described in wave 2. This is in line with previous research in IP/ID personnel in non-pandemic settings, whereby interprofessional power dynamics have been found to can rupture team cohesiveness and trust and increase interpersonal conflict between personnel from different occupational backgrounds (Almost et al., 2016; Keller et al., 2020). Nevertheless, across the waves, occupational categories were used as heuristic shortcut to determine trustworthiness (Davidson et al., 2022; Schilling, 2022). While allowing for quick integration of personnel with relevant skill sets into the team, it also undermined teamwork and integration of deployed personnel without critical care experiences. Despite this finding, professional categories were not always viewed as negative, suggesting that teamwork between IP/ID personnel was often reliant on the individual contribution of a team-member to the team, whereby individual professional skills and experience were used in favor of furthering the team-wide development of shared goals. The study therefore highlights the ambivalent impact of professional categories for teamwork, suggesting that effective teamwork in pandemic healthcare teams requires the reduction of interprofessional power dynamics by transcending prior occupational categories in favor of a new team-wide emergent identity.

Teamwork (and Mental Health) were both further impacted by levels of preparedness, highlighting the need for psychological preparedness of staff as the patient care duties which were most frequently described as being related to psychological strain were those for which participants felt unprepared, including for example patient family liaison, patient distress and deterioration, patient volume and end of life care. More research is therefore needed to assess how different pandemic experiences impact mental health and the differential role of psychological and technical preparedness.

### 4.3 Risk for deployed personnel due to lack of preparedness and social isolation

While many of the above findings are applicable to staff across the occupational spectrum working on COVID wards, the study outlined some important differences between deployed and permanent personnel with regards to the impact on teamwork and cohesion as well as mental health and personal life. The quantitative finding that deployed personnel reported higher levels of impaired work life balance than permanent staff, was supported by the thematic analysis which emphasized that the negative effect of workload on mental health and career intentions appeared to be more pronounced in deployed than permanent personnel. Similarly, higher levels of WLB impairment in HCWs without ICU experience supports the thematic finding that deployed personnel were more likely to discuss not being technically prepared for the work on COVID wards and more likely to deploy involuntarily.

Many deployed personnel, especially those without intensive-care experiences or adequate training, reported lower levels of familiarity with tasks and equipment, which impacted their levels of confidence and sense of contributing to the team, while increasing self-doubt and anxiety. Considering that these personnel were also more likely to discuss ostracization due to a lack of relevant skill sets, this finding is in line with recent work associating lack of technical preparation and unsatisfactory training with higher levels of mental health problems and harmful consequences for people’s job performance (Khajuria et al., 2021; Frenkel et al., 2022). The findings suggest that levels of preparedness may impact upon teamwork, performance, and mental health via lack of group membership.

Likewise, deployed staff were more likely to discuss an absence of social support and opportunities after their deployment and discussed being excluded in meetings or forced to have separate break rooms, which increased social isolation and ostracization. This suggests that in addition to higher risks going into deployment, they faced more issues after deployment, due to not receiving the same care and support that permanent team members enjoyed. As such rapid deployment and redeployment without adequate support risks undermining many of the discussed benefits of cohesion on mental health for this cohort. While this study could not provide correlational data, evidence from other occupational contexts has repeatedly highlighted the increased risk of mental health problems in individually deployed augmentees (Ursano et al., 2017; Cucciare et al., 2020). This suggests that deployed augmentees would benefit the most from interventions that guard against social isolation and ostracization during deployment and the need for specific post-deployment support systems.

### 4.4 Limitations

Due to the inability to conduct observational measures for teamwork during an active pandemic outbreak the study was forced to rely on self-reported descriptions of teamwork, which holds obvious disadvantages compared to other approaches of measuring teamwork in HCWs (Frankel et al., 2007; Kiesewetter and Fischer, 2015; Cooper et al., 2016; Freytag et al., 2019). Likewise, the study relied on a convenience sample of nurses, doctors, allied health professionals and senior leaders who self-referred to participate in the study, thus reducing generalizability of the results. However, considering the large sample size for a qualitative study as well as the diverse participants recruited from NHS trusts across the UK and the comparative character enabling comparison between deployed and permanent staff we believe that the results represent a realistic reflection of the differential experiences and issues faced by personnel working on COVID wards. Another limitation is that as the graph edges are undirected – based on code co-occurrence – the networks must be interpret as relationships without the ability to infer causal statements about directionality. Nevertheless, the results of this exploratory study while providing important lessons for personnel selection, training, co-location, and organizational support during and after a pandemic (Stebbins, 2011; Casula et al., 2021), also inform further research into the differential impact of pandemic deployment on HCWs mental health, interprofessional care delivery, teamwork, and leadership. We therefore propose to test the relationships outlined above in a quantitative dataset. Despite these limitations we believe that the novel approach of utilizing Thematic Network Analysis (Pokorny et al., 2018; Schilling, 2022) to visualize thematically analyzed semi-structured interviews with 75 British HCWs at the frontline of COVID-19 allowed a reproducible visualization of the inherent complexity of qualitative data by highlighting thematic connections and communities which may not be documented using traditional thematic analytic methods.

### 4.5 Implications for practitioners

Participants provided a range of different organizational suggestions and innovations which can aid both managers and leaders during the preparation and response for future pandemics that may require the rapid deployment of personnel from non-intensive care backgrounds into such wards. For example, during rapid upscaling, intervention such as stickers or badges on HCWs PPE, involvement of IP/ID teams into handovers/rounds, designated communal rest areas and fixed intra-group debriefs can increase teamwork, allow information exchange and enable familiarity between colleagues. Similarly, when attempting to increase teamwork and team integration across the wider team, leaders must pay special attention to both the integration of junior or deployed personnel – as these rely more on colleagues for social interactions than senior staff. – and on the management and coordination of social identities capable of transcending prior occupational categories (e.g., ‘we the COVID ward’ vs. ‘them, the physios’). Senior leaders and ward managers should ensure that adequate measures are taken to alleviate stressors (e.g., by employing psychological staff to deal with patient family liaison) while preparing staff for the potential psychological impact of such work. Considering the importance placed on team-based support for the provision of social support and sense-making (e.g., team support groups, social events, debriefs), and the difficulties of many deployed staff to access social support within their teams, it is highly important for hospitals to ensure that all personnel have access to the same team-based support as permanent staff and ensure that organizational support to tackle loneliness and negative mental health consequences are available. Table 4 outlines a range of important organizational, managerial, and mental health suggestions gleaned from the research across the different stages of pandemics, preparation, response, and aftermath.

**Table 4:** Overview of suggestions for better teamwork, team integration, and mental health from the evidence provided.

Overview of suggestions		
Organizational and institutional support	*Integration of pandemic/crisis response* into non-intensive care personnel’s education and periodic training modules prior to deployment in basic skills required.	Pandemic/Crisis preparedness
	*Development and maintenance of a staff roster*, including prior pandemic, infectious disease, or intensive care experience as well as specialized training and skill sets by staff to quickly allocate and deploy during outbreak.	
	*Utilization of skill signifiers*, using stickers or badges on HCWs PPE aids in signifying specific skillsets during high pressure situations and with reduced facial recognition due to PPE (e.g., CPAP trained, intensive care family liasion).	
	*Wide-spread involvement of IP/ID teams* into handovers/ rounds and usage of virtual communication tools to ensure widespread information exchange and development of shared mental models.	Pandemic /Crisis Response
	*Simplicfication and access of health records*, to provide clearly accessible and visible health records in patients rooms to ensure every member of the team can access and contribute to them.	
	*Provision of intra-group debriefs* to facilitate after action review after particular difficult shifts (e.g., with high mortality) and to document clinical procedures and lessons learned.	
	*Integration of designated personnel for specialty taks* (e.g., patient-family liasion) to reduce the burden on frontline HCWs of particular distressing incidents.	
	*Optimisation of designated communal staff rooms and rest areas* to ensure co-location of staff – even during social distancing – as a basis for maintaining information exchange, team cohesion, and familiarity with colleages from all backgrounds.	
Team and ward manager support	*Emphasize visibility and presence on the ward* to facilitate leader legitimacy and be present, and approachable, to all members of the team.	
	*Utilize brief team introductions during handovers*, to ascertain skill-sets of deployed staff and assign responsibilities and tasks based on skill-sets.	
	*Enhance familiarity between personnel*, by de-emphasizing professional categories, but highlighting skill-sets and value to the team and increase personal familiarity.	
	*Establishment of role clarity and vision across the team*, to develop a common understanding of goals and responsibilities and ensure buy-in of all team-members irrespective of prof. Background.	
	*Clearly defined leadership structures*, which empower junior leaders and those with particular professional skill-sets (e.g., family liasion), utilize shared leadership where possible to ensure both managerial and psycho-social support.	
Mental health support	*Re-instate in-person social events, meetings and professional development courses* as quickly as Infection Control guidelines allow, to ensure personnel can benefit from the social interactions with coleagues outside of direct patient contact.	
	*Team-leader support for well-being*, which emphasizes well-being and allows to exhibit mental health awareness, model healthy behaviors and open space to discuss mental health, by regularly checking up on staff to ensure staff well-being and “have their back” vis-à-vis organizational support.	
	*Integration of psychological personnel into frontline teams* was highlighted by many participants as “having been there” was perceived as pivotal for an ability to open up, and allowed the alleviation of immediate concerns and team-wide discussion.	
	*Decompression spaces (e.g., Wobble Rooms)*, allowing staff – either in isolation or with a colleague/ leader – to temporarily retreat, recharge and recuperate after particularly difficult moments.	
	*Mutual support sessions,* with deployed and permanent personnel supported by leaders or psychologists to discuss emotional aspects of their experience, aid in sense-making, find closure, reassure colleagues, and find similarity of experiences (e.g., Schwartz rounds).	Pandemic/crisis Follow up
	*Occupational health support services* (e.g., Psychologists, mental health courses) for personnel to find 1-on-1 support if needed.	

## 5 Conclusion

This study explored permanent and deployed personnel’s experience of COVID work, assessed how interprofessional teamwork was established or maintained despite substantial workplace adjustments and the ways in which participants discussed their mental health during this time. Summarizing such broad issues in one paper inevitably leads to a loss of some of the narrative detail inherent in qualitative data. However, we believe that the novel approach of using thematic network analysis utilized here, offers both the illustration of the inherent complexity of thematic data and a more robust representation of inherent relationships between codes than standard thematic analysis would allow. The presented results show a complicated picture. While the importance of many of the traditional factors associated with the successful delivery of patient care (e.g., team coordination, composition, and team dynamics (4–6)) were highlighted by our participants, they also reported that many of these factors were impeded by pandemic constraints. Hindering ‘business as usual’ by limiting effective collaboration and communication between team-members, depriving leaders of their ability to coordinate and support personnel, and undermining HCW’s access to social and organizational support, pandemic work influenced HCWs ability to effectively work together and cope with stressors both during and after their work on COVID wards. Our research demonstrates that during crisis situations teamwork and successful adaptation to pandemic exigencies may rely on psycho-social, relational, and organizational factors currently under researched. For example, both the relational and structural context of pandemic work (e.g., familiarity with colleagues and tasks, perceived isolation from those outsides of wards, inter-professional hierarchies, (in)voluntary deployment, lack of training) appear to be influencing team-members ability to work effectively with each other, suggesting that successful delivery of care during crisis requires increased attention to the structural consequences of HID clinical guidance. Simultaneously, rapidly developing shared mental models, appraising shared experiences, reducing inter-professional conflict, or creating a socially supportive atmosphere across and beyond occupational boundaries emerge as crucial psycho-social skills when both developing teamwork in rapidly deployed ad-hoc teams and supporting HCW’s ability to cope with pandemic stressors. Considering that leadership was often limited by physical presence on the wards and therefore perceived as shared and attributed to junior leaders, we therefore urgently advocate for the inclusion of training on identity management into team and leadership education. Lastly, the described link between social relationships and participants’ ability to appraise their experience, emphasizes the need for more research on the effect of social group memberships for HCW resilience and continued delivery of care.

## Data availability statement

The datasets generated and analyzed for this study can be found in the supplementary information.

## Ethics statement

The studies involving humans were approved by Oxford Brookes University Research Ethics Committee and the UK NHS Health Research Authority. The studies were conducted in accordance with the local legislation and institutional requirements. The participants provided their written informed consent to participate in this study.

## Author contributions

SS: Conceptualization, Data curation, Formal analysis, Funding acquisition, Investigation, Methodology, Project administration, Software, Validation, Visualization, Writing – original draft, Writing – review & editing. MA: Conceptualization, Data curation, Formal analysis, Investigation, Writing – original draft, Methodology. ZM: Conceptualization, Funding acquisition, Resources, Validation, Writing – review & editing. PC: Conceptualization, Funding acquisition, Validation, Writing – review & editing. MB: Conceptualization, Funding acquisition, Supervision, Validation, Writing – review & editing. VC: Conceptualization, Data curation, Funding acquisition, Methodology, Project administration, Resources, Supervision, Validation, Writing – review & editing.

## Supplementary material

SUPPLEMENTARY FIGURE S1 Psychological Strain and Stressors in all personnel: This graph displays the codes most connected to Psychological Strain and Stressors in all personnel, by filtering Figure 1 using an ego network to only display codes connected to Psychological Strain and Stressors. Additionally, a Fruchterman Reingold layout algorithm was applied to further highlight, through adjacency to the code teamwork, which codes share the most references with teamwork in this group.

SUPPLEMENTARY TABLE S1 Masterfile for all personnel. This matrix table derived from NVivo, shows the number of shared references for the 62 codes used in the thematic network analysis. The association rule lift was calculated and the matrix transformed into a range, which was imported into gephi.

SUPPLEMENTARY TABLE S2 Masterfile for deployed personnel. This matrix table derived from NVivo, shows the number of shared references for the 62 codes used in the thematic network analysis. The association rule lift was calculated and the matrix transformed into a range, which was imported into gephi.

SUPPLEMENTARY TABLE S3 Masterfile for permanent personnel. This matrix table derived from NVivo, shows the number of shared references for the 62 codes used in the thematic network analysis. The association rule lift was calculated and the matrix transformed into a range, which was imported into gephi.

SUPPLEMENTARY TABLE S4 Modularity Clusters and Heatmap of Cluster association of codes across all three graphs. This table shows the calculated modularity cluster of each code across the three graphs. Additionally, a heatmap and summary of overlap is included.

SUPPLEMENTARY TABLE S5 Data Extraction Table: This file contains an example of references which were coded in NVivo against corresponding themes (codes). The table shows both the narrative interpretation of the reference and the different codes that this reference was coded against. The shared occurrence of codes across references is visualized in Figures 1–3.

SUPPLEMENTARY DATA SHEET 1 Interview guide for frontline staff.

SUPPLEMENTARY DATA SHEET 2 Interview guide for leaders.
