# Supplementary figures and images for: “Trust people you’ve never worked with” – A social network visualization of teamwork, cohesion, social support, and mental health in NHS Covid personnel
Source: Front Psychol. 2024 Feb 20;15:1293171. doi: 10.3389/fpsyg.2024.1293171 (PMC10913897; doi:10.3389/fpsyg.2024.1293171)

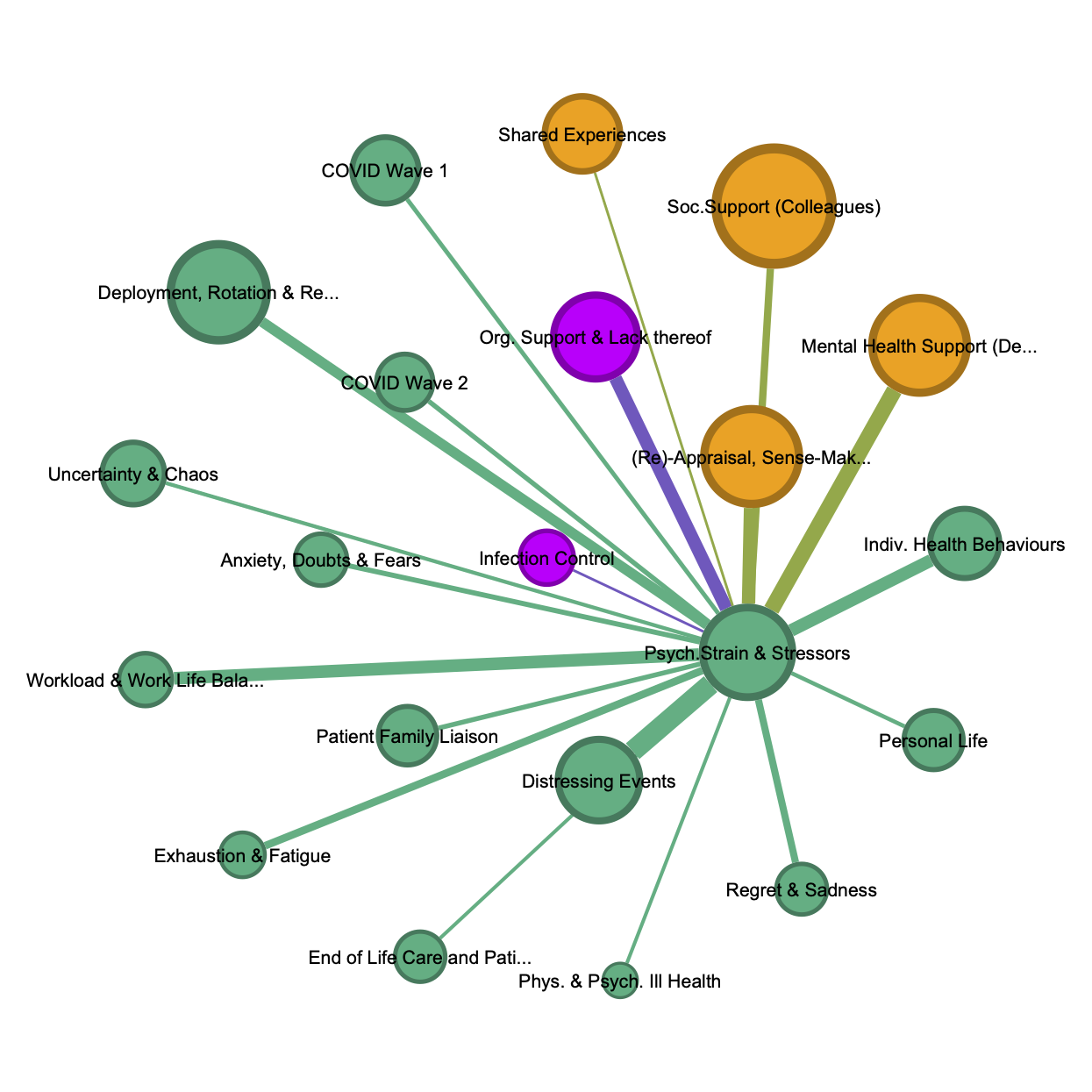

Supplement: SUPPLEMENTARY FIGURE S1 — Psychological Strain and Stressors in all personnel: This graph displays the codes most connected to Psychological Strain and Stressors in all personnel, by filtering Figure 1 using an ego network to only display codes connected to Psychological Strain and Stressors. Additionally, a Fruchterman Reingold layout algorithm was applied to further highlight, through adjacency to the code teamwork, which codes share the most references with teamwork in this group. [file Image_1.tiff]
