# Supplementary material for: “Trust people you’ve never worked with” – A social network visualization of teamwork, cohesion, social support, and mental health in NHS Covid personnel
Source: Front Psychol. 2024 Feb 20;15:1293171. doi: 10.3389/fpsyg.2024.1293171 (PMC10913897; doi:10.3389/fpsyg.2024.1293171)
Supplement: SUPPLEMENTARY DATA SHEET 1 — Interview guide for frontline staff. [file Data_Sheet_1.pdf]

NHS COVID TEAMS STUDY

OXFORD BROOKES UNIVERSITY

Qualitative Interview Guide (Group 1: Patient facing staff)

POSSIBLE WARMUP QUESTIONS:

1.1. Before we get into the questions we've prepared, this interview is about your work on a COVID ward, could you tell us a bit about when you worked there, for how long, and how was it for you to be working in a COVID ward?

1.1.1. OPTIONAL: could you please tell me how you ended up in the COVID team(s) e.g., were you selected, did you change teams or move between different teams?

1.2. Do you feel that your prior work experience prepared you for working on a COVID ward?

1.3. Did you receive any particular training for your COVID deployment, and if what kind?

1.3.1. OPTIONAL: did you find this training to be useful?

TEAM QUESTIONS:

I would like to move on to your particular experience working in a COVID team,

1.4. Could you please describe how you felt when you first entered your COVID team? Was that different to entering a normal team?

1.5. You mentioned earlier that your team referred to [e.g., all the nurses, the interprofessional team] How would you describe the bond between team-members in your team?

1.5.1. OPTIONAL IF POSITIVE: How do you think this bond was created?

1.5.2. OPTIONAL: Did you spend time with team-members outside of direct patient contact, and if, how important do you think this was for bonding?

1.6. During a shift, were there any specific colleagues within your team or the unit you were working in that you would turn more frequently for guidance or support? Why?

1.7. Given the issues around PPE, social distancing, lack of break times, what was the impact on the development of that bond?

1.8. Has this changed at all over the course of the last year.

TEAM-WORK QUESTIONS:

1.9. When you think of your immediate team, I suspect they all are from different backgrounds, given that how did your team develop teamwork?

1.10. Given the different occupational backgrounds and levels of experiences in your team, how did you make sure that you were all working off the same page?

1.10.1. OPTIONAL: Did you develop similar processes or standard operating procedures?

1.11. If you think of a particular shift, can you give me some examples of how good teamwork looked like?

1.11.1. OPTIONAL: How important was trust between team-members for this?

1.11.2. OPTIONAL: How important was communication for your team, and how did you make sure that you could communicate effectively given the issues around PPE, etc.?

1.12. How did teamwork [described by interviewee earlier in the interview] change over the last year within Covid wards?

LEADERSHIP QUESTIONS:

1.13. We are moving on to talk about leadership, could you perhaps tell me who you saw as a leader during your deployment experience?

1.14. How was your relationship with that person and how would you describe their leadership during the COVID experience?

1.15. Do you think the leadership changed at all over the last year within Covid wards?

MODERATING FACTOR QUESTIONS

1.16. During your overall experience working on a COVID ward, can you give us some examples of moments that went particularly well, or moments that did not go well?

1.16.1. OPTIONAL for those that express lack of support/ teamwork/ leadership: Given the circumstances, during that period was there anything that differentiated bad shifts with shifts that went a bit better?

1.17. If you think about some of these moments, what gave you the most support during them?

1.17.1. OPTIONAL: Looking back to your experiences at work over the last year, do you think that relationships with your colleagues or seniors had any impact-positive or negative- on your work-life balance or sense of wellbeing?

1.17.2. You mentioned some innovations that were developed, would you like to see those initiatives continue in your daily professional life after the pandemic. Do you think that they would be useful on a normal day in the ward?

1.18. Did the experience of working with COVID prompt you to alter your future career plans, strengthened your current plans?

1.19. <u>OPTIONAL IF they REDEPLOYED back to old department:</u> Could you please describe how you felt to redeploy back to your old job and how is it to do your old job?

1.20. Have you seen a change in your workload now that your unit has changed back to mostly normal operations, and how do you expect your workload to change in the coming weeks/ months.

1.21. <u>OPTIONAL for family:</u> Did the experience of working on a COVID team impact your family life in any way, for example, was your family very worried?

CLOSING QUESTION:

1.22. Before we finish the interview, is there anything you want to share about your experience during the COVID crisis that we haven't covered?
