# Supplementary material for: “Trust people you’ve never worked with” – A social network visualization of teamwork, cohesion, social support, and mental health in NHS Covid personnel
Source: Front Psychol. 2024 Feb 20;15:1293171. doi: 10.3389/fpsyg.2024.1293171 (PMC10913897; doi:10.3389/fpsyg.2024.1293171)
Supplement: SUPPLEMENTARY DATA SHEET 2 — Interview guide for leaders. [file Data_Sheet_2.pdf]

OXFORD BROOKES UNIVERSITY

NHS COVID TEAMS STUDY

Qualitative Interview Guide Leaders (Group 2: Clinical Directors, Senior Managers and Directors of Nursing)

WARMUP & GENERAL INFORMATION QUESTIONS:

1.1. Could you give us a bit of background information about the chronology of the pandemic response in your hospital? When did it start in your hospital? Was there a significant difference in response between wave 1 and 2?

TEAM STRUCTURE QUESTIONS:

1.2. Did the way you structured your COVID teams differ from how you'd structure a team during routine times?

1.2.1. OPTIONAL: Did you attach/ deploy any additional personnel to your ward/ team?

1.2.2. OPTIONAL: Were you able to keep the core of the team together?

1.3. In light of these limitations, do you think this limited the development of bonds between team-members?

INNOVATION AND LEARNING:

1.4. Do you feel that your previous work experience prepared you for managing during COVID?

1.5. Where did you get information on how to run, manage and structure your personnel?

1.6. Did you share information with other NHS trusts?

1.7. Can you give us some examples of how your team learned in these circumstances? Was there any particular aspect that your team found difficult to learn?

TEAMWORK & LEADERSHIP QUESTIONS:

1.8. Can you describe how your particular team developed teamwork (given that the team-members may not have known each other)?

1.9. Do you think COVID posed particular challenges to effective teamwork within these teams/ wards?

1.9.1. OPTIONAL: Did PPE limit bonding between team-members in any way?

1.9.2. OPTIONAL: HOW did your team develop common standards or common goals?

1.10. Did these challenges impede teamwork and how did you overcome such problems?

1.11. Can you give us some examples of effective teamwork in your team(s)?

1.12. When you think about these examples, what were the most important factors for your team-members to work effectively with each other?

MODERATING FACTOR QUESTIONS

1.13. What support structures did you have in place to support staff during their COVID-deployment and afterwards?

1.13.1. OPTIONAL: Were team-members able to spend any time with each other outside of direct patient contact?

1.14. What have you learned from your experience for the future?

CLOSING QUESTION:

1.15. Before we finish the interview with a few well-being questions, is there anything you want to share about your experience during the COVID crisis that we haven't covered?
